# The association of vitamin D status with oxidative stress biomarkers and matrix metalloproteinases in patients with knee osteoarthritis

**DOI:** 10.3389/fnut.2023.1101516

**Published:** 2023-02-08

**Authors:** Farshad Amirkhizi, Seyed Mojtaba Ghoreishy, Emma Baker, Soudabeh Hamedi-Shahraki, Somayyeh Asghari

**Affiliations:** ^1^Department of Nutrition, Faculty of Public Health, Zabol University of Medical Sciences, Zabol, Iran; ^2^Department of Clinical Nutrition, School of Public Health, Iran University of Medical Sciences, Tehran, Iran; ^3^Cabrini Research, Malvern, VIC, Australia; ^4^Department of Epidemiology and Biostatistics, Faculty of Public Health, Zabol University of Medical Sciences, Zabol, Iran; ^5^Department of Clinical Nutrition, School of Nutritional Sciences and Dietetics, Tehran University of Medical Sciences, Tehran, Iran

**Keywords:** vitamin D, oxidative stress, knee osteoarthritis, clinical, nutrition

## Abstract

**Objective:**

The association of vitamin D status with osteoarthritis (OA) has been demonstrated previously. The current study was performed to examine the association of vitamin D status with oxidative stress markers and matrix metalloproteinases (MMPs) in patients with knee OA.

**Methods:**

This case–control study was conducted on 124 subjects with mild to moderate knee OA and 65 healthy controls. Demographic data was collected from all participants at baseline. Serum levels of vitamin D as well as markers of oxidative stress including malondialdehyde (MDA), total oxidant status (TOS), superoxide dismutase (SOD), oxidative stress index (OSI), paraoxonase-1 (PON-1), glutathione peroxidase (GPX), catalase (CAT), and total antioxidant capacity (TAC) were evaluated for each participant. Furthermore, serum concentrations of MMP-1, MMP-3, MMP-13, and cartilage oligomeric matrix protein (COMP) were measured.

**Results:**

The results of the present study indicated that individuals with vitamin D insufficiency had higher levels of MDA, TOS, SOD, and OSI as well as lower levels of PON-1 and TAC. Based on the linear regression analysis, serum vitamin D levels were inversely correlated with MDA, TOS, SOD, OSI, MMP-1, and MMP-13 and positively associated with TAC levels (*p* < 0.0001). Patients with sufficient vitamin D levels had lower MMP-1 and MMP-13 levels compared to patients with vitamin D insufficiency (*p* < 0.001 and p < 0.001, respectively).

**Conclusion:**

Findings from this study showed a strong association between vitamin D deficiency and increased oxidative stress and MMPs activity in patients with knee OA.

## Introduction

Osteoarthritis (OA), the most common form of arthritis, is a degenerative disorder that disrupts the musculoskeletal system (1). Despite the identification of mechanical, biochemical, and genetic factors, the precise etiology of OA is not entirely elucidated (2, 3). OA is the 11th leading cause of global disability (4), with knee joint involvement being common (2, 5). Knee OA (KOA) affects 3.6% of the total population (nearly 250 million people) worldwide, whereas the prevalence of symptomatic KOA is almost 35% among adults over the age of 60 years (6–9).

In addition to well-known risk factors for KOA, including age, race, sex, prior joint injury, and anatomical joint shape (1, 10), oxidative stress and inflammation also contribute to the pathogenesis of KOA (11–14). Oxidative stress is caused by the overproduction of reactive species, known as pro-oxidants, and the incapability of the antioxidant defense system to scavenge these species, which consequently causes cellular damage and destruction (15, 16). Previous studies have demonstrated the upregulation of reactive species in chondrocytes and cartilage tissue of patients with OA (10, 14, 17, 18). Studies in models of OA have also demonstrated that scavenger enzymes in cartilage are reduced (19, 20).

Matrix metalloproteinases (MMPs), a family of extracellular proteases, are responsible for collagen degradation in the cartilage of OA joints (21). MMPs, especially MMP-1 and MMP-13, cause irreversible destruction of the cartilage matrix through the digestion of type 2 collagen and the subsequent release of matrix proteoglycan from the cartilage (22). The involvement of oxidative stress in MMPs expression has been well documented (23, 24). Accordingly, treatment with antioxidant compounds has been associated with reduced MMP-1 and MMP-9 expression in previous studies (25).

Vitamin D is a well-known main factor for skeletal health and normal bone and cartilage metabolism. It has been proposed to have a protective role against oxidative stress by activating the protective nuclear factor erythroid 2-related factor 2-Kelch-like ECH-associated protein 1 (Nrf2-KEAP1) antioxidant pathway. Therefore, vitamin D may exert its antioxidant effects by up-regulating some of the antioxidant enzymes (26, 27). In addition, it may have potential modulating effects on the expression of certain MMPs (28). Low serum levels of 25(OH)D, the biomarker for vitamin D status, have been associated with increased knee cartilage loss (29), cartilage structural changes (30), quadriceps muscle weakness (31), and more incredible knee pain (32) in patients with KOA. Furthermore, Zhang et al. (33) indicated that the risk of progression of OA doubled in participants with low levels of vitamin D. Very little, however, is known about the biological mechanisms *via* which vitamin D is involved in preventing OA progression (34). To date, findings on the relationship between vitamin D status and oxidative stress biomarkers are mostly related to investigations done on patients with other illnesses, such as multiple sclerosis (35), liver disease (36), and obesity (37). However, the question of whether the conclusions of these patients can also be generalized to patients with KOA remains unanswered. In addition, the available data regarding the relationship between vitamin D status and MMPs in patients with KOA are limited. Therefore, we conducted a case–control study that aimed to evaluate the association between vitamin D status with oxidative stress biomarkers and serum levels of MMPs in patients with KOA.

## Methods

### Patients

The current case–control study was conducted from June to December 2020 on subjects aged 20–60 years who were residing in Zabol County, northeast Iran. The cases were 124 patients diagnosed with mild to moderate bilateral KOA based on the American College of Rheumatology criteria (38) who had attended the hospital or private clinics. The sample size was calculated based on the mean and standard deviation (SD) of serum total antioxidant capacity (TAC) levels as a key variable, obtained from Asghari et al. (39). Considering SD_1_ = 0.5, SD_2_ = 0.3, mean difference (d) of 0.2, α = 0.05, and power = 80%, the sample size was calculated to be 60 subjects. Patients with KOA were categorized as vitamin D sufficient and insufficient based on their serum 25(OH)D levels. Serum 25(OH)D levels of <30 ng/mL and ≥ 30 ng/mL were regarded as vitamin D insufficiency and sufficiency, respectively (40). To assess the KOA severity, they passed a knee radiographic examination based on a Kellgren-Lawrence (K-L) grading system (0 to 4) (41). Mild KOA was considered based on a K-L grade of 1 or 2, and moderate KOA was based on a K-L grade of 3.

Controls were 65 age-and sex-matched healthy subjects with serum levels of 25(OH)D ≥ 30 ng/mL (vitamin D sufficiency) who had attended health centers affiliated with Zabol University of Medical Sciences. Every health center offers primary health care for families within its coverage or reach. All subjects included in the study live close to the border with Afghanistan in southeastern Iran at the latitude of 31○ 2′ 52″N, in a region with 327 sunny days and 4,325 h of sun per year.

Inclusion criteria included a tendency to cooperate in the investigation, the age of 20 to 60 years, and diagnosis with bilateral primary KOA for cases. The exclusion criteria were rheumatic diseases other than KOA, a history of knee surgery and trauma, knee joint replacement, hypertension, cardiovascular diseases, liver diseases, thyroid diseases, diabetes mellitus, kidney dysfunctions, endocrine disorders, inflammatory disorders, and cancer, being lactating or pregnant, using supplements other than vitamin D and calcium 3 months before the research and/or during the research, following a diet for weight reduction, and taking drugs affecting body weight or interfering with vitamin D metabolism, like oral contraceptives, anti-depressants, hormones, anti-psychotics, corticosteroids, and anticonvulsants within the previous 3 months.

Before data collection, the research objectives and protocol were explained to participants before signing informed consent. The research was conducted based on the Helsinki Declaration, and findings were provided following the strengthening of the reporting of observational studies in epidemiology (STROBE) statement for case–control research.

### Anthropometric and physical activity evaluation

All participants were assessed for anthropometric factors. Height measurement was done with no shoes while standing by a fixed non-stretchable tape (precision: 0.1 cm). Weight measurement was done wearing light clothes through a Seca scale to the nearest 0.1 kg. Weight (kg) was divided by squared height (m^2^) for BMI calculation. The International Physical Activity Questionnaire (IPAQ) (42) was applied to assess physical activity levels and then classified as *“light,” “moderate”* and *“heavy”* activity.

### Blood sampling and biochemical assessments

Following a 10-h overnight fasting, a venous blood sample (10 ml) was obtained from the antecubital vein in the morning. Centrifugation of the blood specimens was done (3,000 rpm / 10 min / 4°C for serum acquisition) and kept at −80°C until the assessment.

25(OH)D serum level was evaluated using electro-chemiluminescence immunoassay (ECLIA) on a Roche Elecsys system (Germany) measuring both 25(OH)D3 and 25(OH)D2. The measurement precision and accuracy were monitored by including control specimens (MassCheck controls).

Regarding enzyme activities for each gram of hemoglobin (Hb), the Hb level was evaluated in the hemolysates through a standard kit (Zist chemistry Laboratories, Iran) based on the cyanmethemoglobin technique (Drabkin’s method). The erythrocyte superoxide dismutase (SOD) (EC 1.15.1.1) activity was assessed using a Ransod kit (Cat. No. SD 125, Randox Labs, United Kingdom). Erythrocyte glutathione peroxidase (GPx) (EC 1.11.1.9) activity was measured through the Ransel kit (Cat. No.: RS-504; Randox Laboratories, United Kingdom). The activity of Erythrocytes CAT (EC 1.11.1.6) was evaluated according to Aebi (43), considering the H_2_O_2_ decomposition in phosphate buffer (pH: 7.2) spectrophotometrically at 230 nm. One CAT unit was considered the enzyme amount liberating half of the peroxide oxygen from an H_2_O_2_ solution within 100 s at 25°C. The activity of serum paraoxonase-1 (PON-1) (EC 3.1.8.1) was evaluated through the enzyme-linked immunosorbent assay (ELISA) by an ELISA kit for human PON-1 (SEA243Hu, Cloud-Clone Corp., United States) as instructed.

Serum concentrations of TAC were assessed based on the ferric-reducing antioxidant power approach (44) using a commercial kit (Kiazist chemistry Laboratories, Iran, Cat. No.: KTAC96). Serum concentrations of malondialdehyde (MDA) were measured according to a thiobarbituric acid reactive substance by a commercial kit (Kiazist chemistry Laboratories, Iran, Cat. No.: KMDA96). Serum total oxidant status (TOS) was assessed by Erel’s technique (45) according to the ferrous ion-O dianisidine complex oxidation to ferric ions by specimen oxidants.

The TOS/TAC ratio was applied as the oxidative stress index (OSI). The TAC resulting unit of mmol/L was considered, and the OSI value was determined based on this formula (46): OSI (arbitrary unit) = TOS (μmol H_2_O_2_ equivalent/L)/TAC (mmol Trolox equivalent/L).

Serum concentrations of MMP-1, MMP-3, and MMP-13 were measured based on the immunoassay method using human ELISA kits (Boster Bio-sciences Co., Wuhan, China). Serum concentrations of cartilage oligomeric matrix protein (COMP) were measured by a human ELISA kit as instructed (BioVendor Laboratory Medicine, Inc., Heidelberg, Germany, Cat. No.: RD194080200).

### Statistical analysis

All statistical analyses were done using SPSS software (version 18; SPSS, Chicago, IL, United States). The normality of the data distribution was checked using a Q–Q plot and Kolmogorov–Smirnov test. The results were presented as mean ± standard deviation for normally distributed quantitative data and frequency (percent) for qualitative data. The non-normally distributed data were expressed as the median and interquartile range (IQR). General characteristics were compared using an independent samples *t*-test, Pearson chi-square test, or One-way analysis of variance (ANOVA) with Tukey’s post-test, as appropriate. In addition, between-groups differences in normally and non-normally distributed inflammatory biomarkers were investigated using ANOVA and the non-parametric Kruskal-Wallis test, respectively. To examine the association between serum levels of 25(OH)D and inflammatory biomarkers, multiple linear regression was applied by adjusting for age, sex, BMI, cigarette smoking, physical activity, and use of vitamin D and calcium supplements. *P*-values less than 0.05 were considered significant.

## Results

General characteristics of the study participants according to their vitamin D status are presented in Table 1. In this case–control study, the mean age of 65 healthy controls was 48.1 ± 7.8 years and 47.75% of them were males. Of 124 patients with KOA, 60 patients (48.38%) had sufficient levels of vitamin D (41.8 ± 9.2 ng/mL) and 64 patients (51.62%) had insufficient vitamin D (19.7 ± 5.5 ng/mL) levels. The mean serum levels of vitamin D in healthy controls were not significantly different from KOA patients with sufficient vitamin D levels (42.7 ± 9.6 ng/mL versus 41.8 ± 9.2 ng/mL). In patients with sufficient vitamin D levels, the number of people who took vitamin D and calcium supplements was significantly higher compared to the KOA patients with insufficient vitamin D levels and healthy controls (60% versus 39.1 and 40%, respectively; *p* = 0.031). There were no significant differences in demographic and body composition measures among the study groups (*p* > 0.05).

**Table 1:** General characteristics of healthy controls and patients with knee osteoarthritis according to the vitamin D status.^a^

Variables		Healthy controls (*n* = 65)	Knee OA patients		*P*-value^b^
			Vitamin D sufficient (*n* = 60)	Vitamin D insufficient (*n* = 64)	
Male, *n* (%)		31 (47.7)	26 (43.3)	32 (50.0)	0.753^c^
Age (years)		48.1 ± 7.8	47.8 ± 7.5	50.6 ± 8.6	0.091^d^
Weight (kg)		82.8 ± 13.3	83.4 ± 15.0	84.3 ± 12.4	0.823^d^
BMI (kg/m^2^)		27.6 ± 3.3	28.3 ± 3.2	28.8 ± 3.0	0.101^d^
WC (cm)		94.8 ± 12.3	93.4 ± 13.0	97.0 ± 12.5	0.269^d^
Obesity, n (%)		23 (35.4)	22 (36.7)	26 (40.6)	0.816^c^
Use of vitamin D supplement, n (%)		26 (40.0)	36 (60.0)*	25 (39.1)	0.031^c^
Use of calcium supplement, n (%)		20 (30.8)	31 (51.7)*	22 (34.4)	0.039^c^
Cigarette smoking, n (%)		13 (20.0)	11 (18.3)	15 (23.4)	0.772^c^
Physical activity, n (%)					0.915^c^
	Light	49 (75.4)	47 (78.3)	52 (81.3)	
	MODERATE	9 (13.8)	8 (13.3)	8 (12.5)	
	Heavy	7 (10.8)	5 (8.3)	4 (6.3)	
25(OH)D levels (ng/mL)		42.7 ± 9.6	41.8 ± 9.2	19.7 ± 5.5**	<0.0001^d^
Parathyroid hormone (pg/mL)		49.1 ± 8.3	47.8 ± 7.1	51.0 ± 9.8	0.110^d^
Duration of disease (years)			5.5 ± 2.7	5.9 ± 2.3	0.432^e^

OA, osteoarthritis; BMI, body mass index; WC, waist circumference. Vitamin D sufficient and insufficient was defined as serum 25(OH)D concentrations ≥ 30 ng/mL and serum 25(OH)D concentrations < 30 ng/mL, respectively.

a Values are shown as means ± SD, unless otherwise indicated.

b *P* < 0.05 was considered significant.

c Obtained from Pearson chi-square test.

d Obtained from one-way ANOVA test and Independent sample *t*-test.

e Obtained from independent samples *t*-test.

* Compared to the healthy controls and vitamin D insufficient group.

** Compared to the healthy controls and vitamin D sufficient group.

Table 2 indicates the serum concentrations of oxidative stress markers in healthy controls and patients with KOA according to vitamin D status. KOA patients with vitamin D insufficiency had higher levels of MDA, TOS, SOD, OSI, and lower levels of PON-1 and TAC versus KOA patients with sufficient vitamin D status (*p* < 0.05). The differences in CAT and GPx were not significant among the study groups (*p* > 0.05).

**Table 2:** Oxidative stress markers in healthy controls and patients with knee osteoarthritis according to vitamin D status.

Variables	Healthy controls (*n* = 65)	Knee OA patients		*P*-value^a^
		Vitamin D sufficient (*n* = 60)	Vitamin D insufficient (*n* = 64)	
MDA (nmol/mL)	1.69 ± 0.36	1.87 ± 0.4^***^	2.18 ± 0.5^*^^,^^**^	<0.0001^c^
TOS (μmol H_2_O_2_ Equiv./L)	9.1 (8.0, 10.1)	10.3 (9.0, 16.6)^*^	12.7 (9.8, 17.5)^*^	<0.0001^b^
TAC (mmol/L)	2.1 ± 0.58	1.63 ± 0.47 ^*^	1.42 ± 0.38 ^*^^,^^**^	<0.0001^c^
SOD (U/gHb)	1,134 ± 179	1,156 ± 158	1,237 ± 189^*^^,^^**^	0.003^c^
GPX (U/gHb)	42.7 ± 12.5	44.9 ± 12.3	41.2 ± 11.4	0.217^c^
CAT (K/gHb)	222 (199, 258)	228 (207, 261)	232 (213, 270)	0.261^b^
PON-1 (U/mL)	43.1 (36.2, 58.1)	46.4 (35.3, 62.7)	28.1 (28.0, 48.3)^*^^,^^**^	0.003^b^
OSI	0.54 ± 0.341	0.88 ± 0.53^***^	1.1 ± 0.77^*^^,^^**^	<0.0001^c^

OA, osteoarthritis; MDA, malondialdehyde; TOS, total oxidant status; TAC, total antioxidant capacity; SOD, superoxide dismutase; GPx, glutathione peroxidase; CAT, catalase; PON-1, paraoxonase-1; OSI, oxidative stress index; MMP-1, matrix metalloproteinase-1; MMP-3, matrix metalloproteinase-3; MMP-13, matrix metalloproteinase-13; COMP, cartilage oligomeric matrix protein. Data are expressed as mean ± standard deviation for normally distributed data and median (25th and 75th percentiles) are presented for data not normally distributed (i.e., TOS, CAT, PON-1, MMP-1, MMP-3, MMP-13, and COMP). Vitamin D sufficient and insufficient was defined as serum 25(OH)D concentrations ≥ 30 ng/mL and serum 25(OH)D concentrations < 30 ng/mL, respectively.

a *P* < 0.05 was considered significant.

b Obtained from Kruskal-Wallis test.

c Obtained from one-way analysis of variance (ANOVA).

* Significantly different from the healthy controls (*p* < 0.0001).

** Significantly different from the vitamin D sufficient patients (*p* < 0.001).

*** Significantly different from the healthy controls (*p* < 0.01).

**Figure 1:**
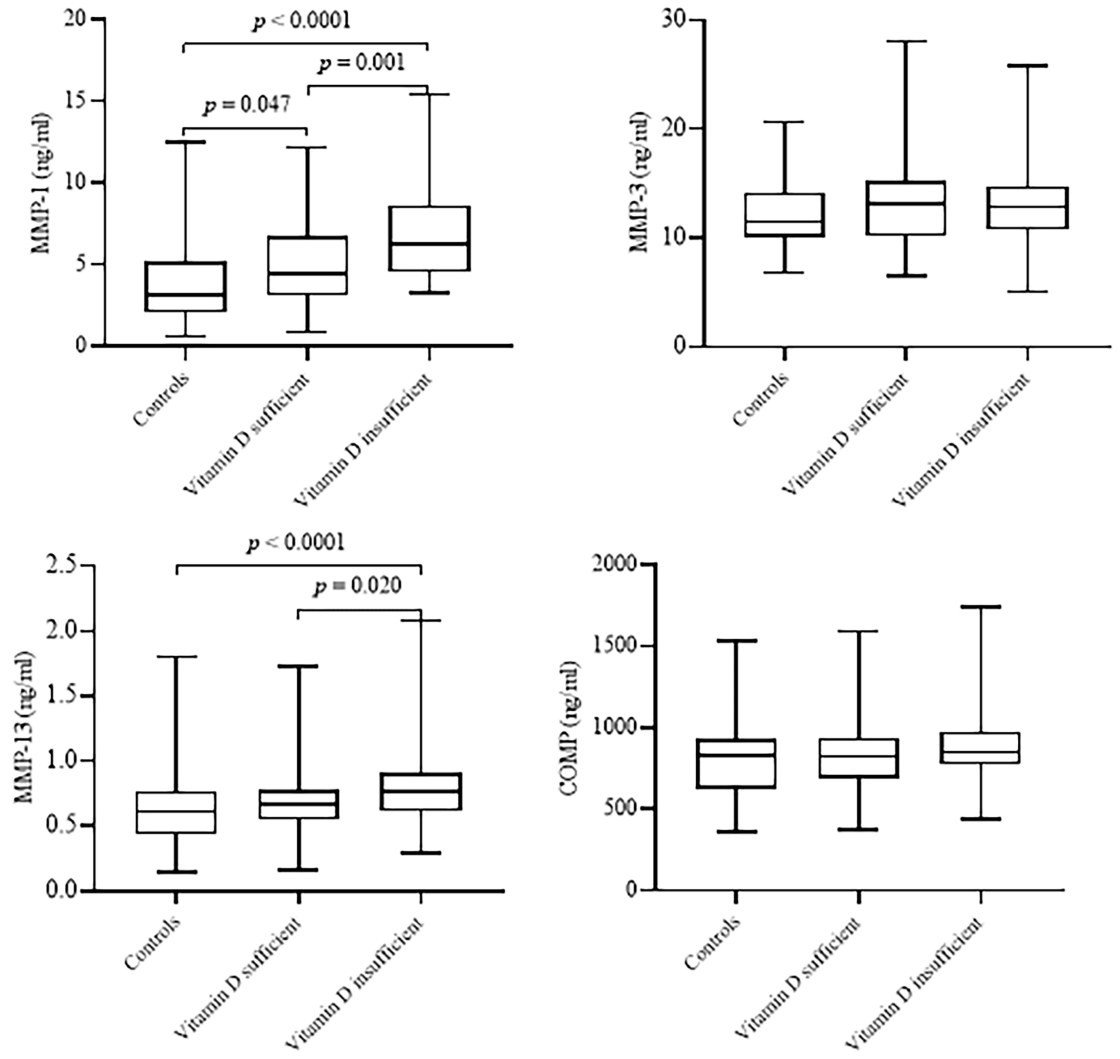
Serum concentrations of matrix metalloproteinases (MMP-1, MMP-3, and MMP-13) and cartilage oligomeric matrix protein (COMP) of healthy controls and patients with knee osteoarthritis according to vitamin D status. Healthy control group, *n* = 65; vitamin D sufficient group, *n* = 60, vitamin D insufficient group, *n* = 64. Vitamin D sufficient and insufficient was defined as serum 25(OH)D concentrations ≥30 ng/mL and serum 25(OH)D concentrations <30 ng/mL, respectively. Data are shown as median (interquartile range). *P*-values obtained from Kruskal-Wallis test. *P* < 0.05 was considered significant.

Figure 1 indicates the serum concentrations of MMP-1, MMP-3, MMP-13, and COMP between healthy controls and patients with KOA according to vitamin D status. Among the three groups, patients with insufficient vitamin D levels had significantly higher MMP-1 and MMP-13 levels compared to the sufficient vitamin D patients (*p* < 0.0001) and healthy controls (*p* = 0.001). No significant differences were seen in MMP-3 and COMP among the study groups (*p* > 0.05).

The results of multiple linear regression analysis investigating the association between serum 25(OH)D and oxidative stress biomarkers and matrix metalloproteinases are shown in Table 3. After controlling for potential confounders including age, sex, BMI, cigarette smoking, physical activity, and use of vitamin D and calcium supplements, serum 25(OH)D levels were inversely correlated with MDA, TOS, SOD, OSI, MMP-1, and MMP-13 and positively associated with TAC (*p* < 0.0001). The associations of 25(OH)D levels with GPX, CAT, PON-1 MMP-3, and COMP levels were not significant.

**Table 3:** Results of multiple linear regression analysis that evaluated the association between serum 25(OH)D with oxidative stress markers and matrix metalloproteinases (*n* = 189).^a^

Variables	Serum 25(OH)D levels		
	B (SE)	β	*P*-value
MDA (nmol/mL)	−0.03 (0.01)	−0.69	<0.0001
TOS (μmol H_2_O_2_ Equiv./L)	−0.17 (0.03)	−0.44	<0.0001
TAC (mmol/L)	0.03 (0.01)	0.72	<0.0001
SOD (U/gHb)	−4.45 (0.96)	−0.33	<0.0001
GPX (U/gHb)	0.10 (0.07)	0.11	0.133
CAT (K/gHb)	−0.23 (0.31)	−0.06	0.450
PON-1 (U/mL)	0.12 (0.11)	0.08	0.300
OSI	−0.03 (0.01)	−0.55	<0.0001
MMP-1 (ng/mL)	−0.10 (0.01)	−0.46	<0.0001
MMP-3 (ng/mL)	−0.03 (0.02)	−0.12	0.101
MMP-13 (ng/mL)	−0.01 (0.01)	−0.30	<0.0001
COMP (ng/mL)	−1.53 (1.14)	−0.09	0.180

MDA, malondialdehyde; TOS, total oxidant status; TAC, total antioxidant capacity; SOD, superoxide dismutase; GPx, glutathione peroxidase; CAT, catalase; PON-1, paraoxonase-1; OSI, oxidative stress index; MMP-1, matrix metalloproteinase-1; MMP-3, matrix metalloproteinase-3; MMP-13, matrix metalloproteinase-13; COMP, cartilage oligomeric matrix protein; B, unstandardized coefficient; S.E., standard error.

a Adjusted for age, sex, BMI, cigarette smoking, physical activity, and use of vitamin D and calcium supplements.

## Discussion

According to our results, KOA patients showed lower vitamin D and TAC levels and higher levels of MDA, TOS, and OSI compared to the healthy controls. The levels of SOD in KOA patients who had vitamin D insufficiency were significantly higher, and the levels of PON-1 were significantly lower compared to the KOA patients with sufficient vitamin D. Significant inverse relationships were seen between serum 25(OH)D and MDA, TOS, SOD, OSI, MMP-1, and MMP-13. However, serum 25(OH)D was positively associated with TAC. As far as we know, the current research is the first case–control study on the association between vitamin D status and oxidative stress markers and MMPs in patients with KOA.

According to the clinical practice guidelines of the Endocrine Society Task Force on vitamin D, the sufficient amount of serum 25(OH)D levels for overall health and bone in normal people should be ≥30 ng/mL. Serum vitamin D concentration of <12 ng/mL is considered vitamin D deficiency which leads to osteomalacia in adults and rickets in children (47). Vitamin D is involved in bone mineralization and bone health (48, 49). It has a protective role against KOA development (33). However, the related mechanisms are not well recognized (34). Inflammation and oxidative stress are involved in the pathogenesis of KOA (11–14). Vitamin D has anti-oxidative and anti-inflammatory activities, making it able to upregulate some of the antioxidant enzymes (26, 27) and modulate the expression of immune-related factors and certain MMPs (28). The associations between 25(OH)D and oxidative stress biomarkers (35–37, 50), as well as some MMPs (51), have been demonstrated. However, these associations have not been investigated in patients with OA.

We showed a lower level of vitamin D and a higher level of oxidative stress biomarkers in KOA patients compared to healthy control subjects, as shown by increased MDA levels, TOS, and OSI, as well as decreased TAC levels. Consistent with the present study, in a 2-year observational study, insufficient levels of vitamin D (<30 ng/mL) was significantly higher among young KOA patients (46.57%) compared to normal cases (24%) (52). Furthermore, Zhang et al. indicated that the risk of progression of OA doubled in participants with vitamin D levels of <15 ng/mL (33). Nonetheless, a meta-analysis reported no relationship between serum 25(OH) vitamin D levels and the KOA risk, however, a significant association was detected between lower vitamin D concentrations and KOA progression (53). The discrepancy between the results of different studies could be due to the different cut-off points defined for vitamin D levels. In a survey of 1,478 children aged 7–11 years, serum 25(OH)D concentrations of <20 ng/mL were defined as vitamin D deficiency, between 20 and 30 ng/mL were defined as vitamin D insufficiency, and > 30 ng/mL were defined as vitamin D sufficiency. In this study a negative relationship between serum vitamin D levels and markers of oxidative stress was declared (54).

In another cross-sectional study, insufficient 25(OH)D levels of <20 ng/mL were associated with increased markers of endothelial activation, oxidative stress, and inflammation in obese children (37). These associations were also reported in patients with chronic hepatitis C (55). All of these findings are in agreement with our results. On the other hand, daily supplementation with 5,000 IU vitamin D in patients with diabetes for 12 weeks did not affect biomarkers of oxidative stress (56).

The relationship between vitamin D and MMPs is observed in some *in vivo* and *in vitro* studies. 1,25-dihydroxycholecalciferol hindered MMPs activity and expression in leucocytes infected with Mycobacterium tuberculosis (57). In addition, vitamin D inhibited the expression and activity of MMP-9 in human lung fibroblasts (58) and MMP-2 in patients with chronic rhinosinusitis (59).

Insufficient vitamin D levels and their effect on degenerative alterations and articular cartilage increase the risk of OA (49, 60). Various mechanisms might describe the association between vitamin D with oxidative stress markers and MMPs. Vitamin D activates Nrf2 gene expression, which regulates some antioxidant and detoxifying enzymes (61–63) and has an inverse correlation with the accumulation of mitochondrial *reactive oxygen species* (*ROS*) (64, 65). Thus, Nrf2 protects cells against oxidative stress (66). On the other hand, it has been shown that 25(OH)D blocks interleukin-1β mediated inhibition of tissue inhibitors of MMPs (58).

It is not known whether oxidative stress markers and MMPs are expressed and produced within the OA joints as well as if the serum levels of these markers could reflect the local levels of them in the synovial fluid of the knee. Further investigation is required to clarify this.

We, for the first time, evaluated the relationship of vitamin D status with oxidative stress biomarkers and MMPs in KOA cases and control cases with sufficient and insufficient vitamin D levels. The subjects were geographically the same, with approximately equal rates of sun exposure. Also, our panel of biomarkers was fairly comprehensive concerning oxidative stress status (MDA, TAC, TOS, SOD, GPX, CAT, PON-1, and OSI) and MMPs (MMP-1, MMP-3, and MMP 13). However, the study also had some limitations that should be considered. Because of the study case–control design, we cannot report cause and effect relationships, and the findings could be biased due to unrecognized confounders, similar to observational studies. We did not use magnetic resonance imaging which would provide more information about the early KOA. Moreover, no radiographic examination of the knees was performed for healthy participants, so asymptomatic KOA may be present in the control group. We could not able to assess the subject’s dietary intakes to study the dietary total antioxidant capacity. Further research is necessary to clarify the mechanisms underlying this association.

We provided further evidence indicating that KOA patients have lower vitamin D levels than control cases without considering taking vitamin D supplements. Also, a reverse association between vitamin D status and serum concentrations of oxidative stress biomarkers such as MDA, TOS, SOD, and OSI, as well as MMP-1 and MMP-13 was detected. TAC was positively correlated with serum vitamin D levels. More studies with prospective designs and large sample sizes are needed to prove these results.

## Data availability statement

The raw data supporting the conclusions of this article will be made available by the authors, without undue reservation.

## Ethics statement

The studies involving human participants were reviewed and approved by Zabol University of Medical Sciences (Code: IR.ZBMU.REC.1398.208). The patients/participants provided their written informed consent to participate in this study.

## Author contributions

FA and SG contributed to study concept, search, data analysis, and drafting of the manuscript. SA and SH-S contributed to data processing, data analysis, and drafting of the manuscript. SA supervised the research. All authors read and approved the final manuscript.

## Funding

This study was supported by Zabol University of Medical Sciences, Zabol, Iran.

## Conflict of interest

The authors declare that the research was conducted in the absence of any commercial or financial relationships that could be construed as a potential conflict of interest.

## Publisher’s note

All claims expressed in this article are solely those of the authors and do not necessarily represent those of their affiliated organizations, or those of the publisher, the editors and the reviewers. Any product that may be evaluated in this article, or claim that may be made by its manufacturer, is not guaranteed or endorsed by the publisher.
